# *In vivo* microbiome and associated immune markers: New insights into the pathogenesis of vaginal dysbiosis

**DOI:** 10.1038/s41598-018-20649-x

**Published:** 2018-02-02

**Authors:** Giuseppina Campisciano, Nunzia Zanotta, Danilo Licastro, Francesco De Seta, Manola Comar

**Affiliations:** 1 SSD of Advanced Microbiology Diagnosis and Translational Research, Institute for Maternal and Child Health - IRCCS “Burlo Garofolo”, Via dell’Istria 65/1, 34137 Trieste, Italy; 2 CBM Scrl-Genomics, Area Science Park, Basovizza, 34149 Trieste, Italy; 3 Medical Sciences Department, University of Trieste, Piazzale Europa 1, 34127 Trieste, Italy; 4 SC of Gynecology - Institute for Maternal and Child Health – IRCCS “Burlo Garofolo”, Via dell’Istria 65/1, 34137 Trieste, Italy

## Abstract

The microbiota fulfils a key role in the training and function of the immune system, which contributes to the symbiosis between the host and complex microbial communities. In this study, we characterized the interplay between vaginal bacteria and local immune mediators during dysbiosis in selected women of reproductive age who were grouped according to Nugent’s criteria. The abundance of *Gardnerella vaginalis* and *Bifidobacterium breve* was increased in the intermediate dysbiotic status, while the presence of a plethora of non-resident bacteria characterized the group with overt vaginosis. In response to these increases, the anti-inflammatory IL1ra and pro-inflammatory IL2 increased, while the embryo trophic factors FGFβ and GMCSF decreased compared to the healthy milieu. A specific pattern, including IL1α, IL1β, IL8, MIG, MIP1α and RANTES, distinguished the intermediate group from the vaginosis group, while IL5 and IL13, which are secreted by Th2 cells, were significantly associated with the perturbation of the commensals *Lactobacilli*, *Gardnerella* and *Ureaplasma*. Summarizing, we postulate that although the dysbiotic condition triggers a pro-inflammatory process, the presence of a steady state level of Th2 may influence clinical manifestations. These results raise clinically relevant questions regarding the use of vaginal immunological markers as efficacious tools to monitor microbial alterations.

## Introduction

The female reproductive tract harbours a symbiotic community that consists of host cells and bacterial communities^1^. Perturbation of the vaginal microbiota is associated with a large spectrum of urogenital diseases that affect many aspects of woman’s lives, including fertility and acquisition of sexually transmitted infections^2–5^. Indeed, the vaginal mucosa is susceptible to the entry of pathogens, where both local immune mediators and commensal microorganisms are the first line of host defence^6–8^.

Bacterial vaginosis (BV) is the most common vaginal dysmicrobism, diagnosed in 20–30% of women of reproductive age, with a prevalence of 50–60% in some high-risk sexual behaviour populations^9^. BV is caused by a reduction of the commensal *Lactobacillus* spp., elevated pH and a concomitant overgrowth of several strict or facultative anaerobic bacteria^10^. Thus, BV is a polymicrobial disease^11–13^ in which a single pathogen is not able to induce the disease. The predominance of *Lactobacillus* spp., as well as the presence of other lactic acid-producing bacteria, appears to be pivotal to counteract the growth of BV-associated bacteria and to reduce the susceptibility to other infections^14–16^.

Although approximately half of BV cases are clinically asymptomatic^17^, an increase of local pro-inflammatory effectors has been observed in women with BV^18,19^. Recent studies have reported the existence of biotic signals that are conducive to a vaginal protective function^20^. In this network, cytokines are interspecies communication molecules that coordinate the interactions of vaginal communities, including the growth of pathogenic microorganisms^21,22^. However, the clinical impact of the modulation of cytokines on vaginal dysbiosis remains to be elucidated.

We profiled the vaginal microbiome and the local immune fluctuation in selected women of reproductive age grouped according to the Nugent’s criteria with the goal of investigating the role of local immune mediators during the vaginal dysbiotic process.

In women with an intermediate Nugent score, we observed that the overgrowth of *Gardnerella vaginalis* was balanced by a high colonization of *Bifidobacterium breve*, which, overall, was able to guarantee a healthy vaginal equilibrium. Conversely, in women with BV, we observed a massive increase of non-resident vaginal species that paralleled a low presence of *G*. *vaginalis*.

The local immune response of the host was specifically stimulated by the vaginal dysbiosis, with a simultaneous increase of the anti-inflammatory IL1ra and the pro-inflammatory IL2 and a decrease of the embryo trophic factors FGFβ and GMCSF. Moreover, a specific pattern of mediators, including IL1α, IL1β, IL8, MIG, MIP1α and RANTES, distinguished the grade of vaginal dysbiosis observed in women with an intermediate Nugent score and in women with overt vaginosis.

Among the tested immune mediators, a significant association of the modulation of specific resident bacterial communities was highlighted for IL5 and IL13. The increase in the concentration of these two anti-inflammatory proteins was accompanied by a depletion of *Lactobacilli* ssp., *G*. *vaginalis* and *Ureaplasma* spp. The concomitant increase of the anti-inflammatory IL5 and IL13, secreted by Th2 cells, and the establishment of a vaginal dysmicrobism suggests a role for the Th2 activation in counteracting the Th1 response and in counteracting the presence of clinical symptoms.

## Results

### Characterization of women with vaginal dysbiosis

To characterize the vaginal dysbiotic process, we collected 62 cervical-vaginal samples from women who were diagnosed according to Nugent’s criteria, including 30 women with a Nugent score of 0–3 (Healthy), 15 women with Nugent score of 4–6 (Intermediate) and 17 women with Nugent score of 7–10 (Vaginosis). The sequencing of the vaginal microbial community yielded 3,496,234 high-quality reads, excluding samples with less than 10,000 reads, with an average of 53,788 reads/sample (range 10,531–190,162). We identified 15,769 operational taxonomic units (OTUs) across all the samples.

### Bacterial diversity and comparisons of alpha diversity between cohorts

Out of the five distinct alpha diversity metrics (within-sample diversity), the Simpson, Shannon, observed species and PD_whole_tree metrics significantly characterized the dysbiotic vaginal microbiome (Table 1) from that of the healthy microbiome, while Chao1 did not exhibit significant variations. Overall, across the five metrics used, the intermediate and vaginosis groups showed higher alpha diversities than the healthy group.

**Table 1:** Comparison of bacterial diversity between cohorts.

	Healthy	Intermediate	Vaginosis	p values
Chao1	400.86 ± 110.87	498.94 ± 147.60	431.37 ± 128.93	0.051; 0.54; 1
Simpson	1.54 ± 0.56	3.55 ± 1.83	3.63 ± 2.270	0.003; 0.003; 1
Shannon	1.25 ± 0.6	2.52 ± 0.86	2.51 ± 0.87	0.003; 0.003; 1
Observed species	154.54 ± 43.08	214.06 ± 49.49	203.17 ± 53.14	0.003; 0.009; 1
PD whole tree	8.36 ± 1.31	10.03 ± 1.56	9.75 ± 1.99	0.003; 0.03; 1

Bacterial diversity values are given as the mean ± standard deviation at a rarefaction depth of 10,000 sequences per sample. Alpha diversity was compared between groups by means of a non-parametric t-test using the compare_alpha_diversity.py script of QIIME. The p values are shown in the last column in the following order: Healthy *vs* Intermediate, Healthy *vs* Vaginosis, and Intermediate *vs* Vaginosis.

**Figure 1:**
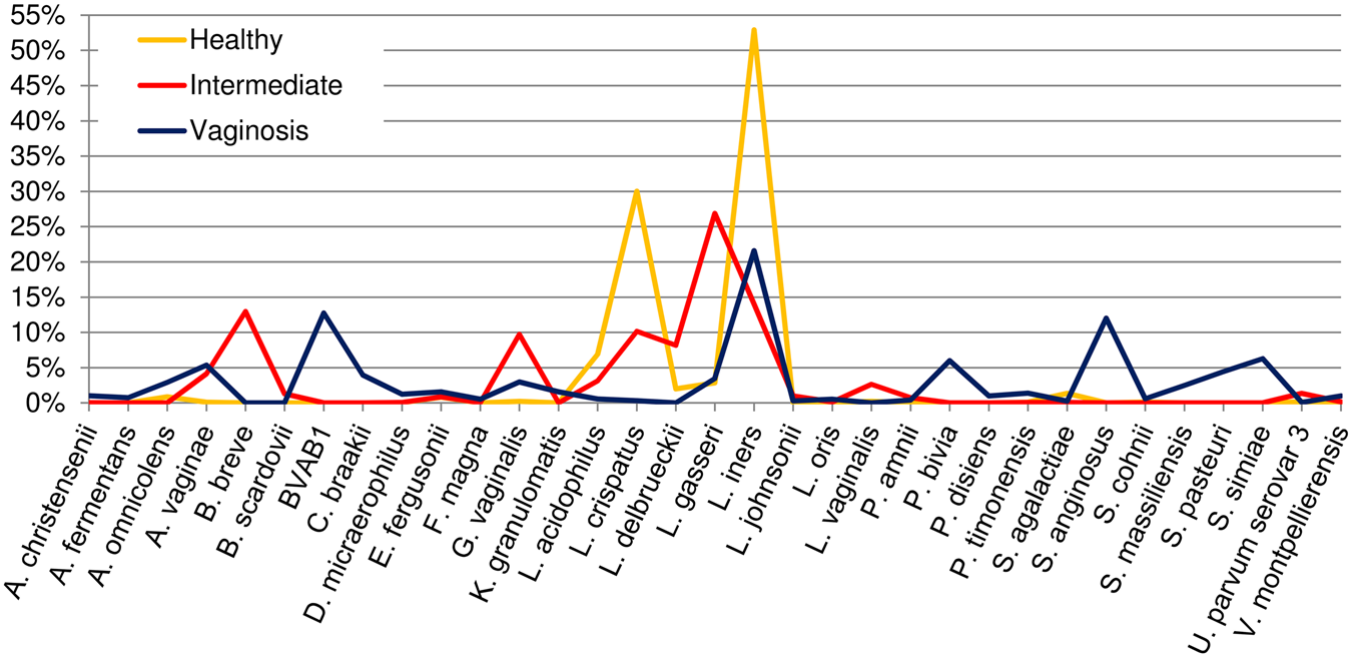
The vaginal bacterial communities from patients with eubiotic and dysbiotic microbiota. The output of plot_taxa_summary.py of QIIME showing the relative abundance of the 33 predominant bacterial taxonomic groups, in alphabetical order, in the studied cohorts: Healthy (Nugent score 0–3), Intermediate (Nugent score 4–6) and Vaginosis (Nugent score 7–10).

When accounting for the bacterial communities in the cohorts (Fig. 1), the intermediate and vaginosis groups showed more heterogeneity among *Lactobacilli* species when compared to the healthy group.

Healthy women exhibited three predominant *Lactobacilli* species, namely *L*. *acidophilus*, *L*. *crispatus* and *L*. *iners* (*Firmicutes*), which were underrepresented in the intermediate and vaginosis groups. *L*. *gasseri* was the most abundant *Lactobacillus* in the intermediate group, while *L*. *iners* was the dominant *Lactobacillus* species in the vaginosis group.

Among the other microorganisms detected, the intermediate group showed an increase of *Gardnerella vaginalis* (*Actinobacteria*) and *Ureaplasma parvum* (*Tenericutes*) compared to the vaginosis and healthy groups. *Bifidobacterium breve* was only detected in the intermediate group.

The vaginosis cohort exhibited a vast change in specific species, which are shown in Table 2.

**Table 2:** BV associated-bacteria.

BV associated-bacteria
*A*. *christensenii (Firmicutes)*
*A*. *fermentans (Firmicutes)*
*A*. *omnicolens (Fusobacteria)*
*A*. *vaginae (Actinobacteria)*
*BVAB1*
*C*. *braakii (Gammaproteobacteria)*
*D*. *micraerophilus (Firmicutes)*
*E*. *fergusonii (Gammaproteobacteria)*
*F*. *magna (Firmicutes)*
*K*. *granulomatis (Gammaproteobacteria)*
*P*. *bivia (Bacteroidia)*
*P*. *disiens (Bacteroidia)*
*P*. *timonensis (Bacteroidia)*
*S*. *anginosus (Firmicutes)*
*S*. *cohnii (Firmicutes)*
*S*. *massiliensis (Firmicutes)*
*S*. *pasteuri (Firmicutes)*
*S*. *simiae (Firmicutes)*
*V*. *montpellierensis (Firmicutes)*

Bacterial identities that were uniquely identified in the Vaginosis cohort.

We assessed which OTUs were discriminative between cohorts, and significant differences of bacterial species were not observed according to the FDR p value.

**Figure 2:**
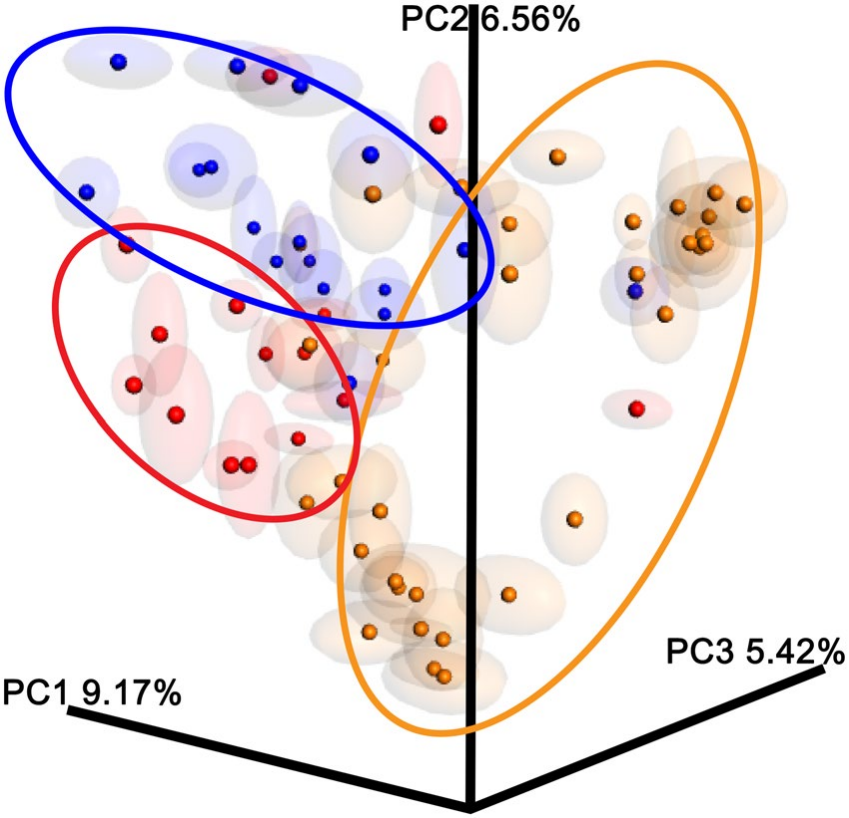
Unweighted UniFrac-based Principal Coordinates Analysis (PCoA). Unweighted UniFrac-based Principal Coordinates Analysis (PCoA) showing the clustering of bacterial communities according to the clinical grouping: Healthy (orange), Intermediate (red) and Vaginosis (blue). Each dot represents a sample. The Emperor PCoA plots were generated from the jackknifed_beta_diversity.py script of QIIME.

**Figure 3:**
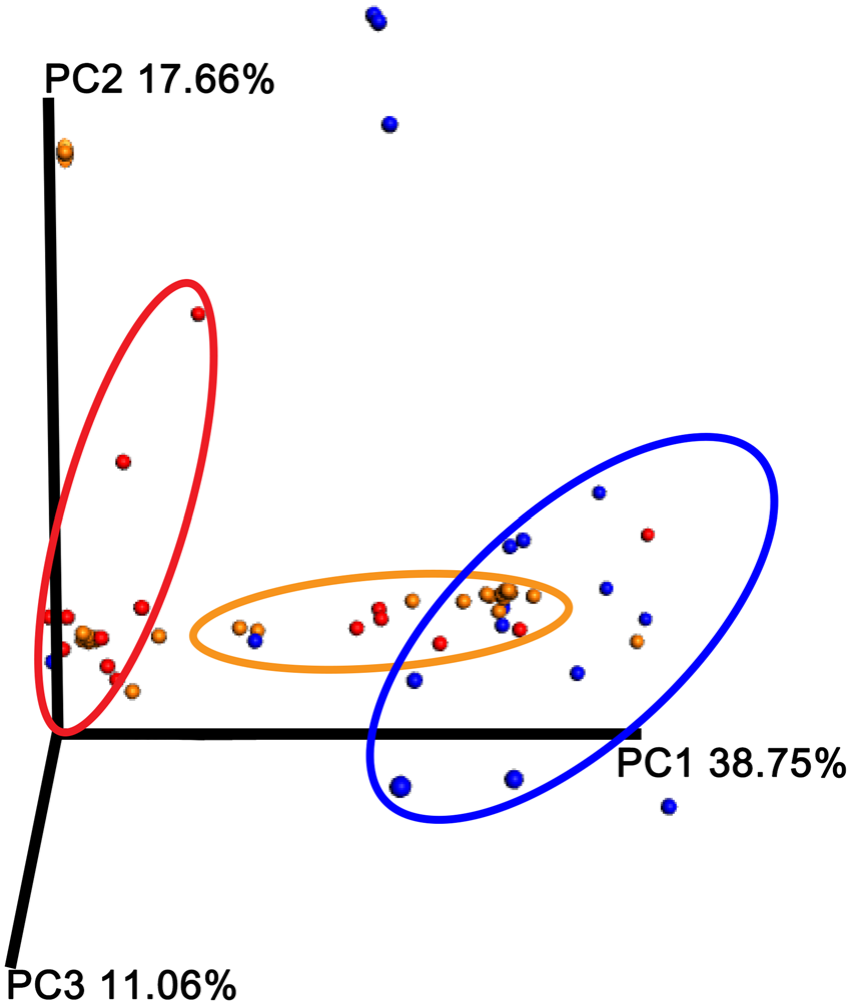
Weighted UniFrac-based Principal Coordinates Analysis (PCoA). Weighted UniFrac-based Principal Coordinates Analysis (PCoA) showing the clustering of bacterial communities according to the clinical grouping: Healthy (orange), Intermediate (red) and Vaginosis (blue). Each dot represents a sample. The Emperor PCoA plots were generated from the jackknifed_beta_diversity.py script of QIIME.

We next compared the overall microbial diversity between groups using the unweighted and weighted UniFrac distance matrices. The results of the β-diversity (between sample diversity comparison) were visualized by a Principal Coordinates Analysis (PCoA). As expected, the bacterial composition of these vaginal communities clustered according to the clinical grouping. Indeed, the analysis of the β-diversity highlights three distinct microbial clusters in the unweighted UniFrac PCoA (Fig. 2). The same clustering is partially confirmed by the weighted UniFrac PCoA (Fig. 3), although it is graphically less evident.

A one-way Analysis of Similarity (ANOSIM) statistical test was applied to the UniFrac distance matrices to test for significant differences according to the clinical grouping. ANOSIM attributed a significant difference to the grouping, shown by the weighted UniFrac (p = 0.001) and unweighted UniFrac (p = 0.001) measures, although the ability of the clinical grouping to explain the differences between cohorts is small (R = 0.31 and 0.21, respectively).

### Correlations between microbiome and immune soluble factors

Forty-eight soluble immune mediators, including cytokines, chemokines and growth factors, were measured in the vaginal samples to address the local immune response to microbial changes, the results of which are shown in Table 3. We observed that an increase of the anti-inflammatory IL1ra and the pro-inflammatory IL2, and a decrease of the embryo trophic factors FGFβ and GMCSF, distinguished the dysbiotic from the eubiotic microbiomes.

**Table 3:** Significant variations in soluble immune factors.

	Healthy	Intermediate	Vaginosis	Healthy *vs* Intermediate	Healthy *vs* Vaginosis	Intermediate *vs* Vaginosis
**cytokines**						
IL1α	62 ± 16	35 ± 6	295 ± 118	0.999	0.08	**0.03**
IL1β	43 ± 28	16 ± 7.5	215 ± 126	0.999	0.064	**0.017**
IL1ra	1.3E5 ± 2.5E4	3E5 ± 4.6E4	1.5E9 ± 1E9	**0.014**	**0.000**	0.605
IL2	1.4 ± 0.44	4.3 ± 0.6	4 ± 0.9	**0.000**	**0.020**	0.999
IL3	121 ± 9	87 ± 10	97 ± 10	**0.028**	0.277	0.999
IL17	12 ± 1.9	5 ± 0.5	7 ± 1	**0.013**	0.209	0.999
IL18	108 ± 21	216 ± 135	1373 ± 593	0.999	**0.000**	**0.001**
MIF	219 ± 113	780 ± 606	2353 ± 890	0.999	**0.019**	0.237
LIF	22 ± 1.3	12 ± 1.5	23 ± 2.7	**0.000**	0.999	**0.002**
SCF	23 ± 3.3	12 ± 2.8	18 ± 2.9	**0.021**	0.999	0.207
TNFα	14 ± 1.7	14 ± 2.3	31 ± 6	0.999	**0.002**	**0.025**
TNFβ	14 ± 1.8	9 ± 0.3	10 ± 0.6	**0.013**	0.999	0.271
**chemokynes**						
IL8	157 ± 26	127 ± 45	351 ± 95	0.646	0.430	**0.047**
MCP1	9 ± 1.6	4.6 ± 0.5	5 ± 0.5	**0.003**	0.061	0.999
MIG	309 ± 119	188 ± 122	488 ± 178	0.08	0.999	**0.016**
MIP1α	1.7 ± 0.3	1.4 ± 0.4	2 ± 0.4	0.561	0.180	**0.012**
RANTES	11 ± 3.7	17 ± 12	24 ± 6	0.406	0.137	**0.005**
**growth factors**						
FGFβ	32 ± 3	17 ± 1.2	18 ± 1.6	**0.008**	**0.019**	0.999
GMCSF	109 ± 4.5	81 ± 6.4	75 ± 4.6	**0.015**	**0.000**	0.593

Data are shown as the mean value (pg/mL) ± standard error of mean. Comparisons between groups were performed using a non-parametric Kruskal-Wallis test. The p values are adjusted for multiple comparisons. Significant p values are highlighted in bold.

A specific pattern of soluble immune mediators distinguished the grade of vaginal dysmicrobism. Specifically, a significant decrease of the proteins IL3, LIF, SCF, TNFβ, MCP1 and IL17 was associated with the intermediate group compared to the healthy group (Table 3). A significant increase of IL18, MIF and TNFα characterized the vaginosis group compared to the healthy group (Table 3). In addition, a panel of cytokines, including IL1α, IL1β, IL8, MIG, MIP1α and RANTES, exclusively differentiated the vaginal inflammatory process of the women who were diagnosed with an intermediate dysmicrobism from those with vaginosis (Table 3).

To evaluate the possible relationship between the local immune response and the bacterial composition, the concentrations of each immune factors were included as variables in the BIO-ENV rank-correlation procedure, where single variables or combinations of variables are selected based on how they best explain differences among samples.

Based on the weighted UniFrac distance matrix, several immune factors showed a high correlation with the different microbial patterns observed among samples. In the dysbiotic microbiota, many of the significantly upregulated cytokines, such as IL18, IL2, IL1ra, MIF, RANTES, TNFα, MIP1α and IL8, were confirmed to correlate with the alteration of the vaginal composition. Other immune factors, such as IL5, IL13, IL6, IL15, IL9, GROα, MIP1β and IFNγ, exhibited a correlation with the altered vaginal milieu, although their amount did not significantly change between cohorts.

Based on the unweighted UniFrac distance matrix, the soluble factors that correlated with the microbiome composition were MIF, IL18, MIP1β, TNFα and IL1ra (Table 4).

**Table 4:** Output of the BIOENV rank-correlation procedure.

	ρ (weighted UniFrac DM)		
IL18	0.273	**IL6**	0.17
IL2	0.264	**MIP1α**	0.166
IL1ra	0.234	**IL15**	0.15
MIF	0.234	**IL8**	0.127
IL5	0.219	**IL9**	0.127
IL13	0.184	**GROα**	0.113
RANTES	0.175	**MIP1β**	0.112
TNFα	0.175	**IFNγ**	0.109
	**ρ (unweighted UniFrac DM)**		
MIF	0.243	**TNFα**	0.102
IL18	0.157	**IL1ra**	0.101
MIP1β	0.137	—	—

Correlations between the 48 immune mediators and both weighted and unweighted UniFrac distance matrices are shown. The table shows the results for ρ ≥ 0.1. The results were generated from the beta_diversity.py output using the compare_categories.py script (metric BIOENV) of QIIME. Abbreviations: ρ: Spearman’s rank correlation coefficient; DM: distance matrix.

Next, we explored whether any individual bacterium correlated with an increase or decrease of specific immune factors. The associations were performed after rarefying the otu_table_biom (depth 10,000 sequences) and using the observation_metadata_correlation.py script with the Fisher_z_transform.

Interleukin IL5 showed a significant correlation (FDR p < 0.01) with *G*. *vaginalis*, *L*. *delbrueckii*, *L*. *acidophilus*, *L*. *johnsonii*, *L*. *gasseri*, *L*. *crispatus* and *L*. *iners*, while IL13 was significantly (FDR p < 0.01) associated with *G*. *vaginalis*, *L*. *delbrueckii*, *L*. *acidophilus*, *L*. *johnsonii*, *L*. *gasseri*, *L*. *crispatus*, *L*. *iners* and *U*. *parvum* serovar 3. The association of other bacteria with the remaining analysed immune factors was not statistically significant when the p value was corrected for the false discovery rate (FDR).

## Discussion

The use of high-throughput sequencing techniques shed new light on the high variability and complexity of the vaginal microbiome. Our *in vivo* results agree with recent studies that have shown that Nugent score, based on the quantification of only three bacterial morphotypes, is not sufficiently informative to clearly define a vaginal dysbiotic status^23^.

Consistent with this issue is the observation of a high colonization by *Bifidobacterium breve*, neglected by Nugent Score, in the vaginal microbiome of women with an intermediate dysmicrobism. *B*. *breve* is able to counteract the suboptimal colonization by the lactate-producing *Lactobacilli spp*.^24^, guaranteeing a healthy vaginal equilibrium through the same mechanism exploited by *Lactobacilli*, that is, the production of lactic acid. It is noteworthy that, by neglecting the presence of all lactate producing bacteria and the identification of *Lactobacilli* species, the Nugent score criteria led to the overestimation of the dysbiotic status and to an unnecessary therapeutic intervention being suggested.

In addition, the Nugent score neglects the dynamic of interspecies communication between specific species of *Lactobacilli* and opportunistic pathogens. In the group of healthy women, *L*. *crispatus*^25^ and *L*. *acidophilus*^26^ inversely correlated with the presence of *Gardnerella vaginalis*, while in women with an intermediate Nugent Score, *L*. *gasseri* negatively correlated with *Atopobium vaginae*^27^, a frequently predominant microorganism in women with BV.

The novelty of our study consists of the *in vivo* demonstration that specific vaginal immune profiles significantly correlate with the microbial composition and clinical manifestation. This finding may help in the diagnosis of BV, predict the recurrence of vaginal dysbiosis and help follow the recovery after treatment. We observed that a subset of pro-inflammatory mediators, typically involved in the chronic inflammation process, synergized with a subset of cytokines that are involved in the switch toward the Th2 immune response. This network likely supports a dysbiotic condition, regardless of the host pro-inflammatory Th1 response, which is normally effective at restoring the eubiosis state.

More precisely, the women with an intermediate Nugent Score showed a significant decrease in several pro-inflammatory cytokines, including IL3^28^ and IL17^13^. This event, together with the poor T cell-stimulating activity of *G*. *vaginalis*^29^, which was predominant in the intermediate group, may explain the absence or weakness of clinical symptoms^11^. Moreover, *B*. *breve*, which was uniquely identified in the intermediate group, can induce Th1 polarization of lymphocytes^30^, supporting their own growth by the production of IL2^31^ and determining the resistance toward the infections. This mechanism may support a biotic balance assuring a healthy vaginal milieu.

In women with vaginosis, a massive pro-inflammatory response was observed, supported by a significant increase of IL18^32^, a constituent of the inflammasome complex^32^. The increase in concentration of other components of the inflammasome, such as IL1β, and the increase of several chemokines, such as IL8, MIG, MIP1α and RANTES, which are involved in the recruitment of leukocytes at the site of inflammation^33^, strongly differentiated women with vaginosis from women with an intermediate Nugent Score. This observation confirms the results of previous *in vitro* studies that showed an immunological shift towards a Th1-dominated profile during episodes of bacterial dysmicrobism^34^.

A new observation that emerged from our study is the significant association between specific commensal microorganisms and the modulation of local cytokines and chemokines, some of which have never been described before. Specifically, IL5 and IL13, which are secreted by Th2 cells^35^ and are known to inhibit both the cell-mediated immune response and several macrophage functions, were statistically associated with *Lactobacilli*, *Gardnerella* and *Ureaplasma* species.

Based on the slight increase of these two cytokines (Table 5) and the depletion of some commensal bacteria (Fig. 1) in women with dysmicrobism, we suggest that the Th2 response is maintained at a steady state level of activation alongside the host Th1 response. The increase of the two anti-inflammatory cytokines is expected to significantly rise in asymptomatic women with an altered microbiome, explaining the absence or the weakness of symptoms. Furthermore, the steady-state Th2 response, together with a blunted Th1 response, could lead to immunologic tolerance causing chronic recurrent vaginal dysbiosis^36^.

**Table 5:** Soluble immune factors related to changes of the microbial composition.

	Healthy	Intermediate	Vaginosis	Healthy *vs* Intermediate	Healthy *vs* Vaginosis	Intermediate *vs* Vaginosis
IL5	0.29 ± 0.02	0.29 ± 0.04	0.35 ± 0.08	0.686	0.999	0.999
IL13	0.82 ± 0.06	0.75 ± 0.07	1 ± 0.19	0.592	0.999	0.580

Data are shown as the mean value (pg/mL) ± standard error of mean. Comparisons between groups were performed using a nonparametric Kruskal-Wallis test. The p values are adjusted for multiple comparisons.

Since IL5 and IL13 are strongly influenced by the alteration of eubiotic conditions, regardless of the specific pathogens causing the alteration of vaginal milieu, they could be further studied as indirect markers of vaginal disorder.

Taken together, our findings provide new *in vivo* insights into how different commensal bacterial species preserve the vaginal symbiotic equilibrium through their interplay with specific local immune mediators. These results raise clinically relevant questions regarding the role of vaginal immunological markers as crucial tool of surveillance of microbial alteration.

## Methods

### Patients and samples

Sixty-two immunocompetent women who fulfilled the inclusion eligibility criteria were included in this study. All women were Caucasian, of reproductive age (32–40 years old), were not pregnant, had no current use of hormonal or barrier contraceptive products, vaginal douching, tobacco or alcohol abuse, were not hospitalized or had systemic use of medication for chronic diseases or antibiotics/probiotics (oral or topical) within the 6 months prior to sample collection, and had no intercourse in the day prior to sampling. Microbiological criteria excluded concomitantly sexually transmitted viral infections, including HSV I-II and HPV.

We identified patients among women that attended the Gynaecology Service of the Institute for Mother and Child Health IRCCS Burlo Garofolo of Trieste, Italy, as outpatients.

According to Nugent’s criteria, 17 women were diagnosed with bacterial vaginosis (Vaginosis, score 7–10) and 15 with an intermediate dysbiotic status (Intermediate, score 4–6) while the remaining 30 women were diagnosed as healthy (score 0–3).

Vaginal samples were collected 7 days before the first day of the menstrual period. Under speculum examination, samples were collected using a 200 mm polyethylene Cervex brush device^37^ (Rovers Medical Devices B.V., The Netherlands) by a single gentle 360° rotation of the cytobrush at the cervical os and were suspended in 1.5 ml of TE buffer. Each sample was divided into 3 (500 µl) aliquots and stored at −80 °C.

### Sample processing and Ion Torrent Sequencing

DNA extraction was carried out using the NucliSENS® easyMAG® system (BioMèrieux, Gorman, North Carolina, USA) with an elution volume of 50 µl. All DNA samples were stored at −80 °C prior to further processing.

A real time EvaGreen PCR (EvaGreen® dye, Fisher Molecular Biology, Waltham, USA) was performed with the degenerate primer 27FYM (5′-AGR GTT YGA TYM TGG CTC AG-3′) and the primer U534R, targeting the V1-V3 region (500 bp) to allow for the construction of rich libraries. A nested PCR, targeting the V3 region^38^, was performed with the primers B338F_P1-adaptor (B338F 5′-ACTCCTACGGGAGGCAGC-3′) and U534R_A_barcode (U534R 5′-ATTACCGCGGCTGCTGG-3′) in conjunction with the IonXpress Barcode Adapter to obtain the 200 bp V3 region template for sequencing analysis^39^. Negative controls, including a no template control, were processed with the clinical samples. The PCR reactions were performed using the Kapa 2 G HiFi Hotstart ready mix 2 × (Kapa Biosystems, Massachusetts, USA), which has robust amplification that is necessary for NGS sequencing^40,41^, and 400 ng/µL BSA, with the following temperature cycling conditions: 5 min at 95 °C, 30 sec at 95 °C, 30 sec at 59°/57 °C, 45 sec at 72 °C and a final elongation step at 72 °C for 10 min.

Quantification of dsDNA was assessed with a Qubit® 2.0 Fluorometer (Invitrogen, Carlsbad, California, USA) and the pooled-library was diluted to a concentration of 100 pM.

Template preparation was performed using the Ion PGM Hi-Q View kit on the Ion OneTouch™ 2 System (Life Technologies, Gran Island, New York, USA) and sequenced using the Ion PGM Hi-Q View sequencing kit (Life Technologies, New York, USA) with the Ion PGM™ System technology.

### Data Analysis

QIIME 1.8.01^42^ was used to process the sequence data. High quality (Q > 20) sequences were demultiplexed and filtered by quality using split_libraries_fastq.py with the default parameters, except for the length parameter (at least 150 bp). Operational taxonomic units (OTUs) were defined at 97% similarity and clustered against the Vaginal 16S rRNA gene Reference Database, which was constructed by Fettweis *et al*.^43^, using open-reference OTU picking^44^ with a uclust clustering tool^45^. Prior to further analysis, singleton OTUs and samples with low sequencing depth were removed (less than 10,000 reads). Chao1, PD whole tree, Shannon, Observed species and Simpson reciprocal metrics were used to assess alpha diversity (within-sample diversity), while beta diversity (between sample diversity comparison) was assessed with weighted and unweighted UniFrac distance matrices^46,47^ and presented with principal coordinates analysis (PCoA). The robustness of the identified clusters was investigated using jackknifing (randomly resampling sequences without replacement). Differences in community composition between cohorts were investigated using analysis of similarity (ANOSIM, 999 permutations) and Kruskal-Wallis test, implemented in QIIME.

Correlations between the immune mediators, for which normalized amounts were written in the mapping file, and both weighted and unweighted UniFrac distance matrices (generated from the beta_diversity.py script) were assessed by the compare_categories.py script (metric BIOENV) of QIIME, exploiting the Spearman rank-order coefficient. A Spearman coefficient close to 1 signifies a highly positive correlation between immune mediators and the distance matrices; a value close to 0 signifies no association; and a value approaching -1 signifies a highly negative correlation.

To survey the association between microbial identities and the increase or decrease of specific immune factors, the observation_metadata_correlation.py script (with the Fisher_z_transform p-value assignment) of QIIME was used. The otu_table_biom was rarefied to a depth of 10,000 sequences/sample and the normalized amounts of the immune markers were written in the mapping file.

The dataset was deposited in the SRA database (PRJNA361297).

### Immune soluble factor quantification

The quantification of soluble immune factors was performed using a recently described platform that is based on a magnetic bead multiplex immunoassay (Luminex, Bio-Plex, BIO-RAD Laboratories, Milano, Italy), which simultaneously detects 48 analytes, including cytokines, chemokines and growth factors^48^.

Briefly, 50 μL of biological samples and standards were added in duplicate into a 96 multiwell plate containing the analyte beads. After incubation for 30 minutes at room temperature and washing, the antibody-biotin reporter was added and incubated for 10 minutes with streptavidin-phycoerythrin. The concentrations of the cytokines were determined using the Bio-Plex array reader (Luminex, Austin, TX). The Bio-Plex Manager software optimized the standard curves automatically and returned the data as Median Fluorescence Intensity (MFI) and concentration (pg/mL). To normalize the results, the total protein concentrations of samples were determined using a Bradford assay (Sigma-Aldrich, St. Louis, MO). Next, all cytokine and chemokine concentrations were normalized to total protein in the sample and were expressed as pg of immune marker⁄mL of total protein.

Stata (v. 13.1) and GraphPad Prism (v. 5) were used for statistical data analysis. The Kruskal-Wallis one-way analysis of variance was used for comparisons between groups. When a significant p-value was observed (p < 0.05), a multiple comparison test was used to determine which groups were different.

### Accession Codes

Sequences have been deposited at NCBI under the accessions PRJNA361297.

### Ethics approval and consent to participate

The protocol used in this study was approved by the Ethics Committee of the IRCCS Burlo Garofolo Institute, Trieste (RC 26/13). All participants provided written informed consent for the experiments involving human participants and gave permission to access their medical records. All experiments were performed in accordance with the Declaration of Helsinki.
